# Intermodulation Component-Based Authentication for Civilian GNSS Signals

**DOI:** 10.3390/s26134047

**Published:** 2026-06-25

**Authors:** Muzi Yuan, Honglei Lin, Jian Liu, Chunjiang Ma, Xiaomei Tang

**Affiliations:** 1 School of Science, National University of Defense Technology, Changsha 410073, China; ymz@nudt.edu.cn; 2 National Key Laboratory for Positioning, Navigation and Timing Technology, Changsha 410073, China; 3 School of Electronic Science and Technology, National University of Defense Technology, Changsha 410073, China

**Keywords:** GNSS signal authentication, intermodulation component, spreading code authentication, constant envelope modulation, spoofing detection

## Abstract

We propose a navigation signal authentication scheme for civilian GNSS receivers that exploits the intermodulation components introduced by constant envelope modulation without relying on modernized authenticatable signals or direct access to authorized spreading codes. Since the product of multiple authorized spreading codes is unpredictable and does not reveal the original codes, the spreading code of the intermodulation component can serve as an authentication feature similar to that of authorized signals. The receiver obtains the intermodulation spreading code via a communication link, then authenticates the GNSS signal by detecting the presence of this code through a correlation-based hypothesis test. We analyze the scheme using the operational GPS L1 signal as an example and compare its performance with Chimera (proposed for GPS), OSNMA (proposed for Galileo), and authorized spreading code authentication in PROSPA. Results show that the proposed scheme achieves robustness and security comparable to Chimera, while avoiding the authorization restrictions associated with authorized codes. Under a 63.82 kbps communication rate and a civilian signal $C/N_{0}$ of 30 dB-Hz, its authentication efficiency exceeds Chimera’s fast channel, with substantially lower data storage requirements.

## 1. Introduction

The global navigation satellite system (GNSS) is widely used in safety-critical applications, which makes its security a pressing concern. Since the Volpe Report [1] identified the vulnerability of GNSS to spoofing, numerous spoofing attacks have been demonstrated against ships [2], vehicles [3], and unmanned aerial systems [4]. These incidents show that spoofing can cause serious harm and highlight the need for effective countermeasures.

Existing anti-spoofing approaches fall into four categories. Observation-based detection uses physical-layer features such as direction of arrival (DOA) [5], inertial measurement unit (IMU) outputs [6] and other receiver-based schemes [7]. Navigation signal authentication detects spoofing by checking for deliberately inserted authenticatable markers in the navigation message [8], spreading code [9], or carrier phase [10]. Communication-assisted authentication uses data exchanged between a receiver and a server to verify signal authenticity [11,12,13,14,15,16]. Authorized signal processing relies on the assumption that spoofers cannot generate encrypted authorized signals. In practice, most communication-assisted authentication methods use authorized spreading codes as the authenticatable feature [17].

However, three limitations persist in current communication-assisted approaches: (i) schemes that depend on authorized spreading codes, such as PROSPA, are restricted to authorized users; (ii) remote-processing architectures, such as ASPIRE, have limited service capacity because each user’s data must be processed individually; and (iii) sample-transmission methods, such as the GPS P(Y)-code correlation approach, suffer from noise in the transmitted samples, which degrades detection performance.

We propose a scheme that uses the intermodulation components of operational GNSS signals for authentication. The key observation is that constant envelope modulation, used to multiplex multiple signal components on operational satellites whose power amplifiers have limited linearity, inherently produces intermodulation components. The spreading code of such a component is the product of the spreading codes of several navigation signal components. When at least two of these are authorized codes, the product is cryptographically unpredictable yet does not reveal the individual authorized codes.

This property rests on the cryptographic design of authorized spreading codes. Taking the GPS M-code as an example, its test-phase version is reportedly generated using AES in a sequential cipher mode, and the Modernized Navstar Security Algorithm (MNSA) in the official version is designed to be no weaker than AES. A standard property of such ciphers is that XORing a cipher-generated keystream with plaintext yields secure ciphertext, and an adversary who obtains only the XOR result cannot recover either input. Since the intermodulation component’s spreading code is the product (XOR-equivalent) of at least two authorized codes, the original codes remain protected, while the product itself serves as an unpredictable authentication feature. This provides authentication capability comparable to that of an authorized signal, but without authorization restrictions.

The main contributions of this work are as follows:

1. We show that the intermodulation components introduced by constant envelope modulation can serve as authenticatable features for civilian GNSS signals, and we derive the conditions under which they can be used without compromising authorized code security.
2. We design a receiver architecture for this scheme and formulate the authentication detection as a binary hypothesis test, enabling quantitative analysis of detection performance.
3. We present three approaches for generating the required spreading code—via the GNSS control segment, via high-gain antenna estimation, and via multikey homomorphic encryption—each trading off real-time availability against authorization requirements.
4. Using the operational GPS L1 signal as a case study, we show that the scheme achieves robustness and security comparable to Chimera, stronger SCER protection than OSNMA, and substantially lower receiver storage requirements than Chimera, without modifying the navigation signal or accessing authorized codes.

The remainder of this paper is organized as follows. Section 2 establishes the system model. Section 3 presents the proposed authentication scheme. Section 4 evaluates the scheme using the operational GPS L1 signal and compares it with existing approaches. Section 5 discusses implications and limitations. Section 6 concludes the paper.

## 2. System Model and Problem Formulation

Throughout this paper, we use the term “authorized signal” to refer to any GNSS signal component whose spreading code is not publicly documented and whose use requires authorization, including GPS P(Y)-code and M-code, BeiDou B3Q and B3A, Galileo PRS and CS encrypted signals, and GLONASS secured signals. The term “authorized spreading code” refers to the spreading code of such a signal component.

This section describes the signal model and establishes the conditions under which intermodulation components can be used for authentication. We use the operational GPS L1 signal broadcast by Block II-F and earlier satellites as a concrete example throughout.

### 2.1. Constant Envelope Modulation

**Figure 1:**
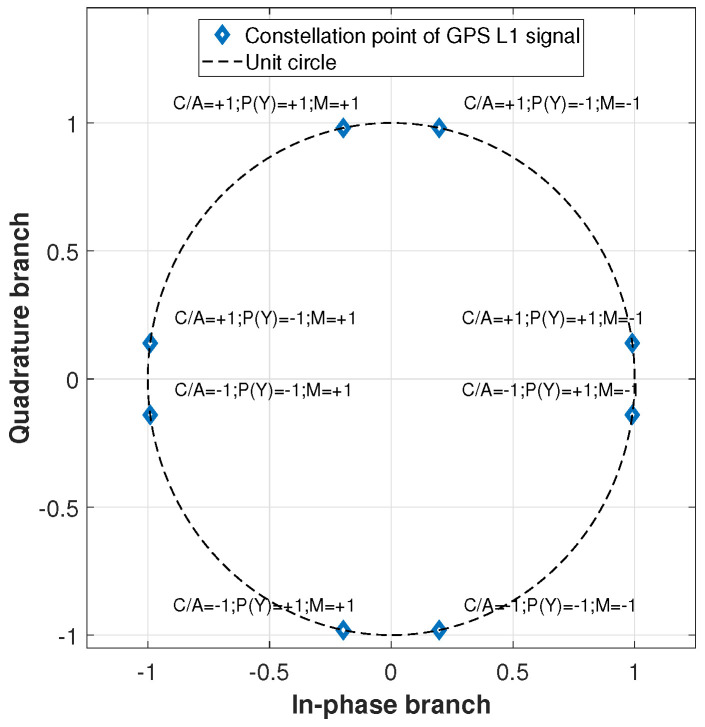
Normalized constellation diagram of the operational GPS L1 signal. The signal contains three navigation signal components—L1C/A, L1P(Y), and L1M—multiplexed by constant envelope modulation. The power ratio is obtained from Partridge et al. [19] and verified through signal observations with a high-gain antenna.

GNSS satellites typically transmit multiple signal components at the same carrier frequency. Some are civilian signals with publicly documented spreading codes, while others are authorized signals with cryptographically generated codes. Because the satellite power amplifier has limited linearity and total transmission power, these components are combined using constant envelope modulation techniques, which keep the signal envelope at a fixed amplitude. Constant envelope modulation introduces additional signal components, known as intermodulation components, to satisfy the power and phase constraints of the individual components. A general model of a constant-envelope-modulated navigation signal is:

(1) $$s\left(t\right)=\sum_{i=1}^{N}s_{i}\left(t\right)+IM\left(t\right)$$

where *N* is the total number of navigation signal components. $IM\left(t\right)$ is the intermodulation component introduced by the constant envelope modulation. $s_{i}\left(t\right)$ is the navigation signal component with index *i*, and can be represented as follows:

(2) $$s_{i}\left(t\right)=\sqrt{P_{i}}C_{i}^{\left(d\right)}\left(t\right)e^{j\left(2\pi f_{c}t+\varphi_{i}\right)}$$

where $j=\sqrt{-1}$ is the imaginary unit, $P_{i}$ is the signal power, and $C_{i}^{\left(d\right)}\left(t\right)$ is the spreading code modulated with the navigation message. The constant envelope property requires that the signal satisfy:

(3) $$\forall T_{1}<T_{2},\frac{1}{T_{2}-T_{1}}\int_{T_{1}}^{T_{2}}{\left|s\left(t\right)\right|}_{f_{c}=0}^{2}dt=P_{s}$$

where the constant $P_{s}$ is the total power of the GNSS signal. The constant envelope characteristic causes the constellation points of a GNSS signal to lie on the unit circle. For example, the operational GPS L1 signal contains one civilian signal component, L1C/A, and two authorized signal components, L1P(Y) and L1M [18]. The normalized constellation diagram for this signal is shown in Figure 1.

Multiple algorithms can achieve constant envelope modulation, such as majority logic [20], coherent adaptive subcarrier modulation (CASM) [21], and phase-optimized constant-envelope transmission (POCET) [22]. GPS Block IIR-M and Block II-F satellites use the CASM technique to achieve constant envelope modulation [19]. Most constant envelope modulation techniques introduce intermodulation components; thus, such components are inherent to multi-signal constant-envelope signals.

### 2.2. Intermodulation Components

If a navigation signal comprises *N* signal components, the maximum number of intermodulation components $N_{IM}$ introduced by constant envelope modulation is given by

(4) $$N_{IM}=\sum_{i=2}^{N}\left(\begin{array}{c} N\\ i \end{array}\right)=\sum_{i=2}^{N}\frac{N!}{i!\left(N-i\right)!}=2^{N}-N-1$$

The intermodulation component with index *i* can be represented as follows.

(5) $$s_{IM_{i}}\left(t\right)=\sqrt{P_{IM_{i}}}C_{IM_{i}}\left(t\right)\exp\left[j\left(2\pi f_{c}t+\varphi_{IM_{i}}\right)\right]$$

where $P_{IM_{i}}\geq 0$ and $\varphi_{IM_{i}}\in \left[0,2\pi \right)$ are the power and phase of the intermodulation component, respectively. $C_{IM_{i}}$ is the spreading code of the intermodulation component. It is the product of the spreading codes of at least two navigation signal components, which can be expressed as

(6) $$C_{IM_{i}}\left(t\right)=\prod_{j=1}^{∥K^{\left(i\right)}∥}C_{K_{j}^{\left(i\right)}}^{\left(d\right)}\left(t\right)$$

where $\left\{K^{\left(i\right)}\right\}$ is the index set, indicating which navigation signal component spreading codes compose the spreading code of the intermodulation component with index *i*. $∥K^{\left(i\right)}∥\geq 2$ is the total number of elements in the index set, and $K_{j}^{\left(i\right)}$ is the *j*-th element in the index set. The aggregate intermodulation signal can then be expressed as

(7) $$IM\left(t\right)=\sum_{i=1}^{N_{IM}}s_{IM_{i}}\left(t\right)=\sum_{i=1}^{N_{IM}}\sqrt{P_{IM_{i}}}\left[\prod_{j=1}^{∥K^{\left(i\right)}∥}C_{K_{j}^{\left(i\right)}}^{\left(d\right)}\left(t\right)\right]\exp\left[j\left(2\pi f_{c}t+\varphi_{IM_{i}}\right)\right]$$

In practice, the number of intermodulation components may be fewer than the theoretical maximum $N_{IM}$. In the operational GPS L1 signal broadcast by Block IIR-M and Block II-F satellites, the authorized signal components L1P(Y) and L1M have opposite phases and are in quadrature with the civilian L1C/A component [19]. The civilian L1C/A component and the authorized L1P(Y) component share the same LNAV navigation message. The CASM technique introduces a single intermodulation component, yielding the following baseband signal model:

(8) $$\begin{array}{cc} s\left(t\right) & =\sqrt{P_{P(Y)}}C_{P(Y)}\left(t\right)D\left(t\right)\exp\left(j\varphi_{P(Y)}\right)\\ \; & +\sqrt{P_{M}}S_{BOC}\left(t\right)C_{M}\left(t\right)D_{M}\left(t\right)\exp\left(j\varphi_{M}\right)\\ \; & +\sqrt{P_{C/A}}C_{C/A}\left(t\right)D\left(t\right)\exp\left(j\varphi_{C/A}\right)\\ \; & +\sqrt{P_{IM}}C_{C/A}\left(t\right)C_{P(Y)}\left(t\right)S_{BOC}\left(t\right)C_{M}\left(t\right)D_{M}\left(t\right)\exp\left(j\varphi_{IM}\right) \end{array}$$

where $P_{P(Y)}$, $P_{M}$, $P_{C/A}$, and $P_{IM}$ are the power of L1P(Y), L1M, L1C/A, and the intermodulation component. $C_{P(Y)}\left(t\right)$ and $C_{M}\left(t\right)$ are cryptographically generated authorized spreading codes. $S_{BOC}\left(t\right)$ is the square-wave subcarrier of binary offset carrier (BOC) modulation in the L1M component. $C_{C/A}\left(t\right)$ is the public civilian spreading code of the L1C/A component. $D\left(t\right)$ is the LNAV navigation message shared by L1P(Y) and L1C/A [18]. $D_{M}\left(t\right)$ is the navigation message of the L1M component. $\varphi_{P(Y)}$, $\varphi_{M}$, $\varphi_{C/A}$, and $\varphi_{IM}$ are the fixed phase offsets of L1P(Y), L1M, L1C/A, and the intermodulation component. Table 1 lists the power and phase of each component in the operational GPS L1 signal.

**Figure 2:**
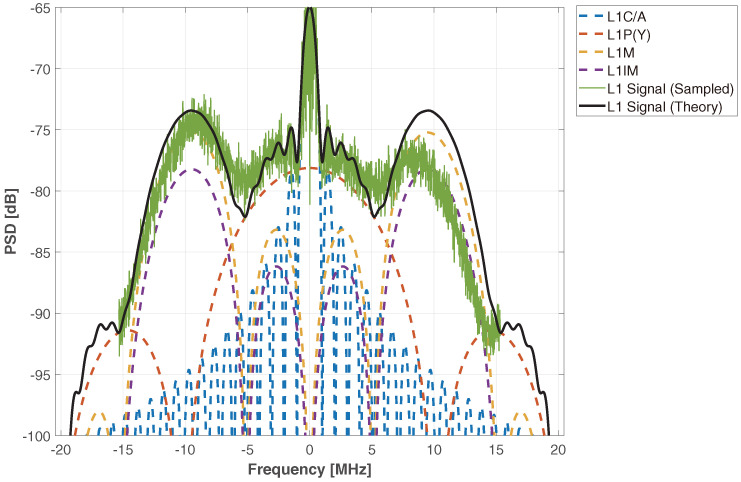
Power spectral density of the operational GPS L1 signal broadcast by Block IIR-M and Block II-F satellites. The green curve shows the observed spectrum from data collected by a parabolic high-gain antenna at the authors’ affiliation. The signal was sampled on 24 July 2021 from PRN-03 of the GPS constellation. The asymmetry on the right side of the observed spectrum is caused by receiver filtering in that frequency band. The dashed and solid black curves are computed from the theoretical model using the power ratios reported by Partridge et al. [19].

The power spectral density of the signal is shown in Figure 2. The observed power spectrum from satellite PRN-03 (Block II-F), sampled by a parabolic high-gain antenna, confirms the correctness of the GPS signal composition described in Section 2.2.

### 2.3. Signal Authentication via Intermodulation Components

Not all intermodulation components are suitable for signal authentication. If all factors that constitute the spreading code of an intermodulation component are civilian codes, the resulting sequence is predictable and cannot serve as an authentication feature. If the product contains only one authorized spreading code and the remaining factors are publicly known civilian codes, the intermodulation component’s spreading code is, at the authorization level, equivalent to that authorized code—authentication via such a component inherits the same authorization restrictions as direct authorized code authentication.

The intermodulation components useful for authentication are those whose spreading codes are the product of at least two different authorized spreading codes. Because different authorized spreading codes are generated independently, their product is independent of each original code. Recovering any of the original authorized codes from the product is cryptographically infeasible, while the product itself remains unpredictable. Thus, when the spreading code of an intermodulation component contains at least two authorized spreading codes, it provides authentication capability comparable to that of an authorized signal component while eliminating authorization constraints.

A subtle point concerns whether revealing the XOR of two encrypted spreading codes weakens authorized signal security. An attacker who observes the XOR result can determine whether the two encrypted chips have the same or opposite polarity, which reduces the $C/N_{0}$ required for chip guessing. However, because encrypted chips are publicly broadcast and no longer confidential once transmitted, there is no mandate to prevent access to the sequence. Therefore, this reduction in guessing difficulty does not materially compromise authorized signal security.

Based on the structural analysis of the constant envelope modulation and the resulting intermodulation components, the intermodulation component in the operational L1 signal broadcast by GPS Block IIR-M and Block II-F satellites can be modeled as follows.

(9) $$\begin{array}{cc} s_{IM}\left(t\right) & =\sqrt{P_{IM}}C_{C/A}\left(t\right)C_{P(Y)}\left(t\right)S_{BOC}\left(t\right)C_{M}\left(t\right)D_{M}\left(t\right)\exp\left(j\left(2\pi f_{c}t+\varphi_{0}\right)\right)\\ \; & =\sqrt{P_{IM}}C_{C/A}\left(t\right)C_{P}\left(t\right)C_{W}\left(t\right)S_{BOC}\left(t\right)C_{M}\left(t\right)D_{M}\left(t\right)\exp\left(j\left(2\pi f_{c}t+\varphi_{0}\right)\right) \end{array}$$

where the P(Y) code is obtained by multiplying the 10.23 Mcps public P-code with an approximately 511.5 kcps cryptographically generated W-code, i.e., $C_{P(Y)}\left(t\right)=C_{P}\left(t\right)\;C_{W}\left(t\right)$. This intermodulation component contains exactly two authorized spreading codes (W-code and M-code), whose product $C_{W}\left(t\right)\;C_{M}\left(t\right)$ is an unpredictable sequence suitable for authentication. The code rate of this product is the higher of the two, namely 5.115 Mcps of the M-code.

Note that the W-code and M-code have different chipping rates. This creates a risk: an attacker who correctly guesses the polarity of a single W-code chip can, with 50% probability, guess the M-code sequence during the same chip interval. To mitigate this, the server should distribute only one higher-rate spreading code chip per chip interval of the lower-rate code. For instance, the server distributes only the M-code chip at the start of each W-code chip period. This prevents leakage of the higher-rate sequence during the lower-rate chip interval. Although this makes the authentication spreading code non-contiguous, the feasibility of authentication is unaffected: since authentication is performed after the signal has been stably tracked, sufficient detection energy can be accumulated by summing correlation results over multiple coherent integration intervals. Because the receiver does not need to cache signal samples outside the authenticated code window, this code-provisioning strategy does not alter the detection performance relative to the continuous-code case.

The proposed scheme authenticates the spreading code, which protects the ranging measurement against spoofing. Navigation message authentication, which is a separate function, can be achieved more easily over the same communication link because of the low message data rate. The scheme therefore focuses on the more challenging problem of ranging authentication.

The principle described above for the GPS L1 signal is not limited to this signal. Other GNSS signals that carry at least two restricted-access components at the same carrier frequency—such as GPS L2 and potentially certain Galileo signals—can also support this authentication method.

## 3. Proposed Authentication Scheme

This section presents the proposed intermodulation component authentication scheme. We describe the receiver architecture, formulate the authentication detection problem as a binary hypothesis test, and introduce three complementary approaches for generating the required intermodulation component spreading code.

### 3.1. Overall Structure

**Figure 3:**
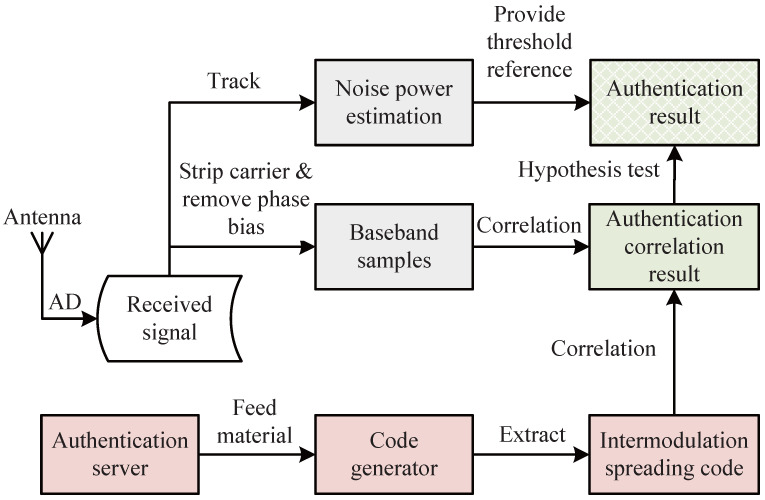
Overall structure of an authenticatable GNSS receiver for intermodulation component-based authentication.

An intermodulation component authentication receiver can be constructed by augmenting a conventional GNSS receiver with an authentication correlator and an authentication spreading code generation/reception unit coupled to the tracking module. A typical architecture is shown in Figure 3.

The receiver first receives and tracks the civilian component of the GNSS signal. Using the tracking channel observations, the carrier and signal dynamics can be stripped, and the intermodulation component at the corresponding phase can be extracted. The noise power can be estimated through a noise branch in the tracking module. At the same time, the receiver obtains the code generation material from the authentication server through the communication link, and the code generator uses this material to produce the authentication spreading code (i.e., the intermodulation component spreading code). The receiver then correlates the authentication spreading code with the baseband samples. The correlation result is compared against a constant false alarm threshold derived from the noise power estimate provided by the noise branch, yielding a hypothesis test for signal authentication. Through this process, spoofing detection is achieved.

The core prerequisite of the proposed authentication scheme is the availability of a data communication link between the receiver and the server. This prerequisite is readily satisfied by modern receivers equipped with communication capabilities, such as mobile smart devices.

### 3.2. Hypothesis Testing for Authentication Detection

Authenticating the signal reduces to detecting whether the unpredictable spreading code (the product of authorized codes) is present in the received signal. This can be formulated as a binary hypothesis test:

(10) $$\begin{array}{cc} H_{0} & :s\left(t\right)=s_{p}\left(t\right)+n\left(t\right)\\ H_{1} & :s\left(t\right)=s_{p}\left(t\right)+s_{u}\left(t\right)+n\left(t\right) \end{array}$$

where $s_{p}\left(t\right)$ is the predictable (civilian) signal component, $s_{u}\left(t\right)$ is the unpredictable intermodulation component, and n(t) is additive white Gaussian noise. We use a matched-filter correlator $f\left(s\left(n\right)\right)=\sum s_{l}\left(n\right)s\left(n\right)$, where $s_{l}\left(n\right)$ is the locally reconstructed intermodulation component sample. Under each hypothesis, the detector output follows a different distribution.

Assuming the intermodulation spreading code is orthogonal to the civilian code, the correlation noise reduces to Gaussian white noise. The detector output distributions under the two hypotheses are then:

(11) $$\begin{array}{cc} p\left(f\left(s\left(n\right)\right)|H_{0}\right) & ∼N\left(0,\sigma_{n}^{2}\right)\\ p\left(f\left(s\left(n\right)\right)|H_{1}\right) & ∼N\left(\sqrt{P_{s}},\sigma_{n}^{2}\right) \end{array}$$

where $P_{s}$ is the signal power. $\sigma_{n}^{2}=kT/T_{coh}$ is the noise power. $T_{coh}$ is the coherent accumulation time of the correlator. According to the model discussed above, the constant false alarm detection threshold $Th\left(P_{FA}\right)$ for signal authentication can be expressed as follows.

(12) $$Th\left(P_{FA}\right)=\sigma_{n}Q\left(P_{FA}\right)$$

where $Q\left(x\right)=\int_{x}^{+\infty }\frac{1}{\sqrt{2\pi }}\exp\left(-\frac{1}{2}t^{2}\right)dt$ is the right-tail probability function of the standard normal distribution. The noise power can be estimated using the noise branch of the tracking loop.

### 3.3. Authentication Spreading Code Generator

The intermodulation component spreading code is derived from the product of multiple authorized spreading codes and is pseudorandom with no predictable period. This section describes three methods for generating the intermodulation component spreading code. Other methods beyond these three can also produce the required codes. Different generation methods affect the feasibility and performance of authentication; however, none should compromise the security of the underlying authorized spreading codes.

#### 3.3.1. Generation via Authorized Devices and Transmission

**Figure 4:**
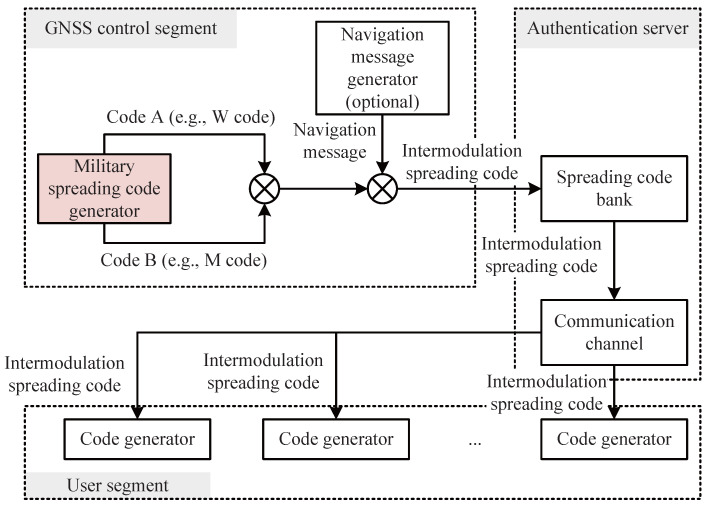
System connection for obtaining the authentication spreading code of the intermodulation component through the GNSS control segment.

Service centers cooperating with the GNSS constellation operator can obtain the intermodulation component spreading code directly from the control segment. A data link connects the GNSS control segment to the service center, which receives the required product of authorized spreading codes generated by authorized devices in the control segment. This approach is illustrated in Figure 4.

In this approach, only the authorized spreading code generator at the control segment requires authorization and management. The unpredictable sequence can be distributed in advance of the signal broadcast time without strict authorization constraints, enabling the receiver to perform real-time authentication without buffering signal samples.

The key advantage is that authorized users receive the intermodulation component spreading code ahead of time, enabling real-time processing with locally stored codes and reducing implementation cost. The disadvantage is that if an attacker can also obtain the spreading code in advance, they could construct spoofed signals. This necessitates user identity management, which limits the scheme’s large-scale deployment. This code-provisioning method is therefore best suited for highly trusted users, such as secure financial infrastructure and critical power-grid nodes.

#### 3.3.2. Estimation via High-Gain Antennas and Transmission

**Figure 5:**
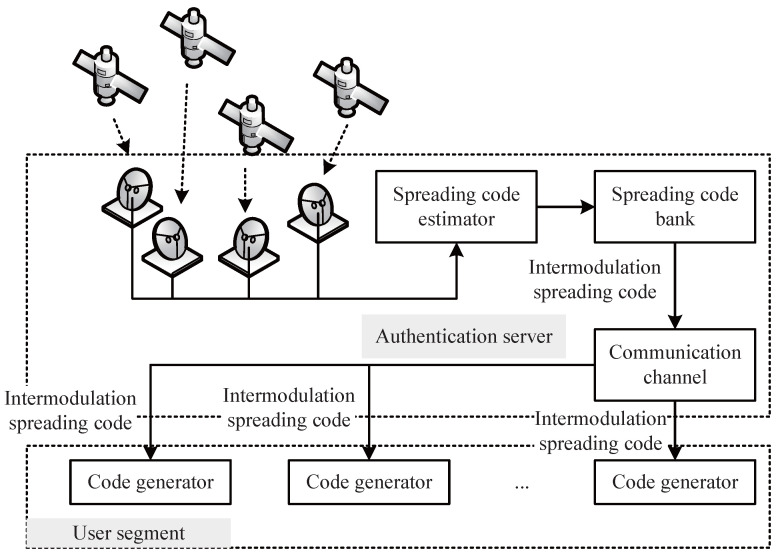
System connection for obtaining the authentication spreading code of the intermodulation component through estimations from high-gain antenna observations.

For users independent of the GNSS control segment, a high-gain antenna observation system can be deployed to observe the authorized signal components and estimate the authorized spreading codes. The estimates of multiple authorized codes are then multiplied to produce the intermodulation component spreading code, which is transmitted to the user. This approach is illustrated in Figure 5.

The advantage of this approach is that no authorization is required: the M-code and P(Y)-code can be observed simultaneously and transmitted to the user, providing strong authentication capability. However, the unpredictable sequence obtained lags behind the GNSS signal, so the receiver must buffer signal samples and complete authentication after receiving the spreading code. This introduces a time delay between signal reception and authentication—a characteristic common to many authentication schemes. For example, OSNMA requires reception of an entire navigation message frame, Chimera’s slow channel requires collection of complete digital signatures, and PROSPA requires waiting to receive authorized spreading codes. The feasibility of the proposed scheme under this generation method is comparable to these established approaches, with the added advantage of requiring no modification to the navigation signal and no impact on authorized spreading code security.

#### 3.3.3. Generation via Multikey Homomorphic Encryption

A forward-looking approach for generating intermodulation component spreading codes exploits multikey homomorphic encryption techniques [23]. If different authorized signal components employ the same multikey homomorphic cryptosystem to generate spreading codes, the cryptosystem can produce a homomorphic key group satisfying the following property:

(13) $$enc_{k_{a}}\left(d_{1}⊕d_{2}\right)=enc_{k_{b}}\left(d_{1}\right)⊕enc_{k_{c}}\left(d_{2}\right)$$

where $enc_{k}\left(d\right)$ denotes encryption of data *d* under secret key *k*, and ⊕ is the bitwise XOR operation. The key $k_{a}$ for generating the intermodulation component spreading code can be derived from the keys of the two authorized spreading codes $k_{b}$ and $k_{c}$ via a key derivation function:

(14) $$k_{a}=KD\left(k_{b},k_{c}\right)$$

The service center can directly distribute the homomorphic key corresponding to the intermodulation component spreading code to the receiver. The receiver generates the product of the authorized spreading codes using this key without accessing the individual authorized codes. This approach offers the highest efficiency among the three methods. However, it requires that authorized signal spreading codes be generated by an algorithm compatible with the multikey homomorphic cryptosystem, which is not the case for operational navigation signals. This method is therefore forward-looking.

## 4. Performance Evaluation

This section evaluates the proposed scheme along four dimensions: robustness, security, efficiency, and cost. Robustness measures the receiver’s ability to reliably detect spoofing. Security assesses the difficulty for an attacker to compromise the authentication mechanism. Efficiency captures how frequently authentication can be performed. Cost encompasses the implementation overhead for receivers, the GNSS constellation, and supporting infrastructure.

We take the operational GPS L1 signal as a concrete example and compare our scheme with three representative authentication schemes that are close to practical deployment: Chimera (proposed for GPS) [9], OSNMA (proposed for Galileo) [8], and authorized spreading code authentication in PROSPA [14] of the EXPLORERS program [15].

Table 2 summarizes the key parameters used in the performance comparison.

### 4.1. Robustness

**Figure 6:**
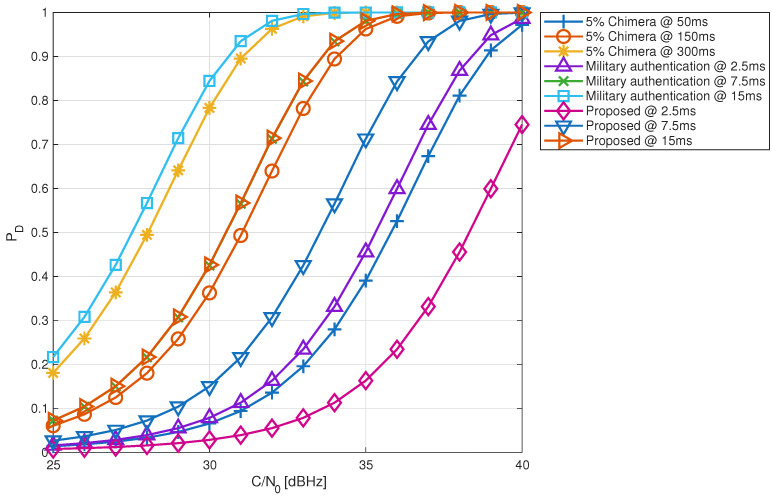
Detection probability of different authentication schemes. The false alarm probability is set to $P_{FA}=10^{-3}$.

**Figure 7:**
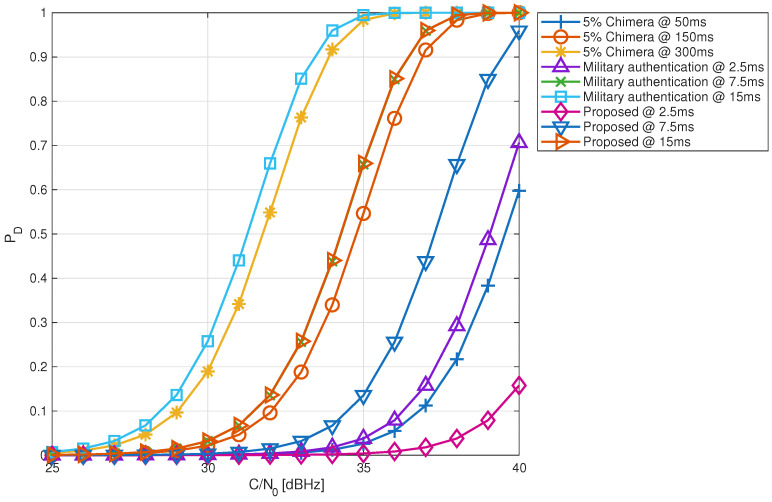
Detection probability of different authentication schemes. The false alarm probability is set to $P_{FA}=10^{-6}$.

Robustness is measured by the detection probability at a fixed false-alarm probability—the probability of correctly detecting a spoofed signal when the probability of falsely flagging an authentic signal is held constant. Using the detection theory from Section 3.2, the detection probability as a function of $C/N_{0}$ is shown in Figure 6 and Figure 7.

The performance gap between intermodulation component authentication and L1M authorized spreading code authentication is mainly due to the power difference: the intermodulation component in the operational GPS L1 signal has a power of −161 dBW, versus −158 dBW for the L1M authorized component. Consequently, the $C/N_{0}$ required for equivalent detection performance is approximately 3 dB higher for the intermodulation component.

Since both the proposed scheme and Chimera are spreading code authentication methods, their robustness can be related through an equivalence relationship. Let DF denote the duty factor of Chimera authentication, and let $\alpha_{IM}=P_{IM}/P_{L1C}$ be the relative power of the intermodulation component to the civilian signal component hosting Chimera markers. The equivalence between the signal collection time of Chimera $L_{Chimera}$ and that of the proposed scheme $L_{IM}$ is:

(15) $$L_{IM}=L_{Chimera}\times \frac{DF}{\alpha_{IM}}$$

For example, with a Chimera marker duty factor of 5% and the intermodulation component being 2.5 dB weaker than the civilian signal component, the ratio of signal collection times required for the same detection probability is approximately 0.0889. The suggested Chimera collection time of 150 ms thus corresponds to approximately 13.33 ms for the proposed scheme.

### 4.2. Security

Security concerns the scheme’s resistance to two classes of attacks: cryptographic analysis and security code estimation and replay (SCER) [24].

#### 4.2.1. Cryptographic Analysis Attack

Without access to the cryptographic keys, the intermodulation component spreading code is unpredictable, so an attacker cannot directly synthesize a counterfeit signal. A cryptographic analysis attack attempts to predict future spreading code values from past observations. Using a secure cipher with an adequate key length makes such prediction computationally infeasible. Since the authentication spreading code inherits the structure of the authorized code, its resistance to cryptographic analysis is the same as that of the underlying authorized signal.

#### 4.2.2. Security Code Estimation and Replay (SCER) Attack

An SCER attack is designed specifically to bypass navigation signal authentication. If successful, the attacker can produce a spoofed signal that passes authentication verification.

In an SCER attack, the attacker first receives and tracks the authentic GNSS signal. The authentication symbols are treated as information-bearing elements, and an estimator is set up to recover their values. These estimates are then used to construct a spoofed signal [24].

Unlike simple re-transmission spoofing, SCER attacks can achieve negative latency—the spoofed signal can precede the authentic one while carrying the recovered authentication symbols, thereby passing authentication verification. The attack proceeds as follows: before the estimation is complete, random values fill the corresponding position in the spoofed signal. Once the estimator recovers the symbol, the estimated value is inserted for the remainder of that symbol’s duration, and the spoofing signal power is raised. The effectiveness of this attack depends critically on the symbol duration: a longer symbol width allows more accurate estimation and greater temporal freedom for forgery.

SCER attacks pose a significant threat to authentication signals. In the absence of additional countermeasures, the primary defense is to minimize the unpredictable symbol width (USW) in the authentication signal. USW is therefore the principal metric for evaluating SCER resistance.

Table 3 shows the USWs of the proposed scheme, Chimera, OSNMA, and the authorized signal L1M. The table shows that the proposed scheme achieves a SCER protection level similar to that of Chimera and authorized signals, and higher than that of OSNMA, which relies solely on navigation message authentication.

### 4.3. Efficiency

Authentication efficiency is assessed mainly through the time to first authenticated fix (TTFAF) and the time between authentications (TBA). These are related as follows:

(16) $$TTFAF=\frac{TBA}{2}+\bar{TBA}$$

The relationship between TBA and average TBA is given by

(17) $$\bar{TBA}=\frac{TBA}{P_{D}}$$

The communication link data rate directly affects TBA and, consequently, TTFAF. Assuming the receiver requires a signal collection time of *N* to perform robust authentication and the communication link rate is $R_{b}$, the TBA can be expressed as:

(18) $$TBA=\left\{\begin{array}{cc} \frac{R_{u}}{R_{b}}-1, & R_{b}<R_{u}\\ 0, & R_{b}\geq R_{u} \end{array}\right.$$

**Figure 8:**
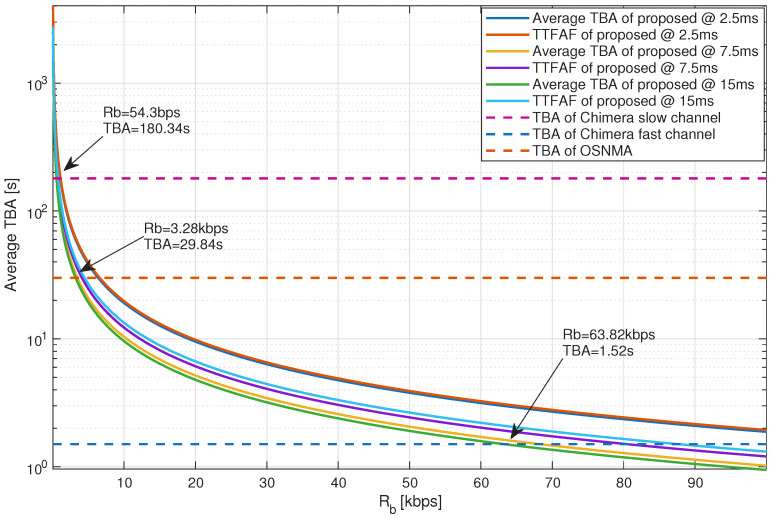
TTFAF and average TBA of the proposed scheme as functions of the communication rate, evaluated at a civilian signal $C/N_{0}$ of 30 dB-Hz.

Here, $R_{u}$ is the data rate of the unpredictable spreading code, which is 5.115 Mcps for the operational GPS L1 signal. The efficiency of intermodulation component authentication is identical to that of L1M authorized spreading code authentication. Figure 8 shows the TTFAF and average TBA of the proposed scheme as functions of the communication rate, evaluated at a civilian signal $C/N_{0}$ of 30 dB-Hz.

As shown in Figure 8, a communication rate of 0.54 kbps is sufficient to achieve a TBA of 180 s, matching Chimera’s slow channel. Matching the 1.5 s TBA of Chimera’s fast channel requires at least 63.82 kbps. These values are evaluated at a civilian signal $C/N_{0}$ of 30 dB-Hz with $P_{FA}=10^{-3}$. Compared with OSNMA, the proposed scheme achieves an average TBA of 30 s (the designed TBA of OSNMA) at a communication rate of 3.28 kbps.

### 4.4. Cost

The main implementation cost of intermodulation component authentication consists of communication link overhead and receiver storage overhead (for caching either signal samples or authentication spreading codes). Because the scheme requires no modification to the navigation signal, it does not affect civilian signal reception or processing. The communication overhead has been analyzed in the Section 4.3; Section 4.4 focuses on storage requirements. Since the storage overhead is identical to that of authenticating via the L1M authorized signal component, we compare the proposed scheme only with Chimera.

**Figure 9:**
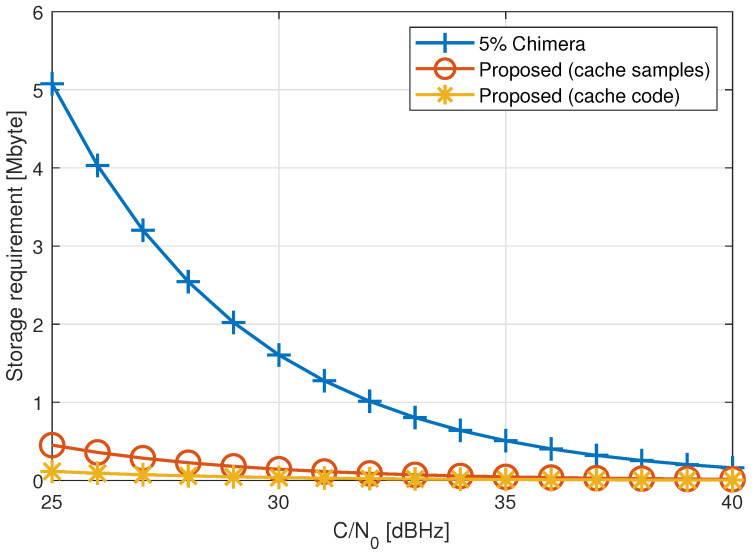
Storage requirement of a receiver with 2-bit quantization and 10 MHz sampling rate. A detection probability of 99.5% is achieved at $P_{FA}=10^{-3}$.

The storage overhead arises from caching either signal samples (when the authentication spreading code lags behind the GNSS signal) or the authentication spreading code (when it is received in advance). This tradeoff is analogous to that in Chimera. Figure 9 shows the storage requirement for a receiver with 2-bit quantization and a 10 MHz sampling rate, achieving a detection probability of 99.5% at $P_{FA}=10^{-3}$.

As shown in Figure 9, the storage requirement of the proposed scheme is substantially smaller than that of Chimera. This advantage arises because Chimera’s authentication markers are sparsely distributed (a typical duty factor of 5%), so only a small fraction of the signal collection time contributes to authentication. In contrast, although the intermodulation component has lower signal power, all spreading code chips within the collection interval participate in authentication.

## 5. Discussion

The proposed intermodulation component authentication scheme offers a pathway to authenticatable navigation for civilian receivers without requiring either modernized GNSS signals or access to authorized spreading codes. Several aspects merit further discussion.

### 5.1. Applicability to Other GNSS Constellations

The proposed method is applicable to any GNSS signal where at least two authorized signal components are multiplexed at the same carrier frequency using constant envelope modulation. We analyze the applicability across the four major GNSS constellations.

GPS. Both the L1 and L2 frequencies carry two authorized signal components—P(Y)-code and M-code—satisfying the applicability condition. The GPS L1 signal, used as the primary example in this paper, is one such case.

BeiDou (BDS). The BDS-3 B3 band (1268.52 MHz) carries the open service signal B3I alongside two authorized signal components, B3Q and B3A [25]. Since B3Q and B3A share the same carrier frequency, constant envelope multiplexing produces intermodulation components whose spreading codes contain at least two authorized codes. The proposed scheme is therefore applicable to BDS B3 signals.

Galileo. The E6 band (1278.75 MHz) carries the PRS signal (E6-A, government-authorized access) alongside the Commercial Service signals (E6-B and E6-C), which employ encrypted spreading codes. Their constant envelope multiplexing produces intermodulation components suitable for authentication.

GLONASS. Traditional GLONASS uses frequency division multiple access (FDMA), where each satellite transmits on a slightly different carrier frequency; the intermodulation mechanism described in this paper does not apply in that configuration. The newer GLONASS-K2 satellites transmit CDMA signals on L1 and L2 with both open service and secured components at the same carrier frequency [26]. However, only one secured component per frequency has been observed to date. Moreover, K2 currently transmits FDMA and CDMA signals through separate antenna arrays, so constant envelope multiplexing between them is not required. Future GLONASS satellites are expected to adopt a single navigation antenna, which will require constant envelope multiplexing of FDMA and CDMA signals through a common amplifier chain [26]. In that configuration, intermodulation between the FDMA secured signal and the CDMA secured signal would produce components suitable for the proposed method. The applicability to GLONASS is therefore prospective, pending the deployment of single-antenna satellites.

Table 4 summarizes the applicability analysis.

The specific performance parameters (e.g., required signal collection time, storage requirements) would need to be recomputed for each signal based on the power ratios of the intermodulation components.

### 5.2. Limitations and Future Work

The proposed scheme has several limitations that warrant further investigation. First, the authentication efficiency depends on the communication link data rate; receivers without broadband connectivity may experience a longer TBA. Second, the code-generation approach via high-gain antenna estimation introduces a latency between signal reception and authentication, although this latency is comparable to that of existing schemes such as Chimera’s slow channel. Third, the multikey homomorphic encryption approach, while offering the highest efficiency, requires authorized signals to adopt a compatible cryptosystem; this is not the case for current constellations. For operational signals, the control-segment and high-gain-antenna approaches remain the practical options.

The experimental results reported in this paper are based on real GPS L1 signal observations collected using a parabolic high-gain antenna (see Section 2.2). A natural next step is to deploy a prototype server infrastructure and conduct long-term field trials to evaluate the scheme under diverse spoofing scenarios and environmental conditions.

## 6. Conclusions

This paper proposed a communication-assisted authentication scheme for civilian GNSS receivers that exploits the intermodulation components inherently introduced by constant envelope modulation. Unlike existing approaches, the scheme requires neither modernized authenticatable signals nor direct access to authorized spreading codes. The principal findings are as follows:

1. The intermodulation component produced by constant envelope modulation of multiple authorized signals carries an unpredictable spreading code that can serve as an authentication feature, provided the code is the product of at least two authorized spreading codes. Revealing the product does not materially compromise the security of the underlying authorized codes.
2. The proposed receiver architecture supports detection of the intermodulation component spreading code through binary hypothesis testing, with performance analytically characterized under varying $C/N_{0}$ conditions.
3. Performance evaluation on the operational GPS L1 signal demonstrates that the scheme achieves: (a) robustness and security comparable to Chimera; (b) stronger SCER protection than OSNMA; (c) authentication efficiency exceeding Chimera’s fast channel at a communication rate of 63.82 kbps and a civilian signal $C/N_{0}$ of 30 dB-Hz; and (d) substantially lower receiver storage requirements than the suggested Chimera configuration.

These results establish intermodulation component authentication as a viable and practical approach for protecting civilian GNSS signals against spoofing attacks, particularly in applications where receivers have communication capabilities but cannot be granted access to authorized spreading codes.

## Figures and Tables

**Table 1:** Power and phase of signal components in the operational GPS L1 signal broadcast by Block IIR-M and Block II-F satellites. The power ratio is obtained from Partridge et al. [19]; the reference power of the civilian signal is specified in the GPS interface specification [18].

Component Label	Power (dBW)	Phase (rad)
L1P(Y)	−161.5	0
L1M	−158.0	π
L1C/A	−158.5	π/2
L1IM	−161.0	π/2

**Table 2:** Key parameters of each authentication scheme used in the performance comparison.

Parameter	Proposed	Chimera	OSNMA	PROSPA
Authentication signal	IM (GPS L1)	L1C/A marker	—	E1-A PRS snippet
Authentication code rate	5.115 Mcps	1.023 Mcps	—	2.5575 Mcps
Duty factor	100%	5%	—	100%
$P_{FA}$	$10^{-3}$/$10^{-6}$	$10^{-3}$/$10^{-6}$	—	$10^{-3}$/$10^{-6}$
Civilian $C/N_{0}$	30 dB-Hz	30 dB-Hz	30 dB-Hz	30 dB-Hz

**Table 3:** USWs of the proposed scheme, Chimera, OSNMA, and the authorized signal L1M.

Scheme	USW
Chimera proposed for GPS	0.9775 μs
OSNMA proposed for Galileo	4 ms
Authorized signal authentication	0.1955 μs
Proposed authentication scheme	0.1955 μs

**Table 4:** Applicability of the proposed authentication scheme to the four major GNSS constellations.

Constellation	Frequency	Authorized Components	Status
GPS	L1, L2	P(Y)-code, M-code	Applicable
BeiDou	B3 (1268.52 MHz)	B3Q, B3A	Applicable
Galileo	E6 (1278.75 MHz)	E6-A (PRS), E6-B/C (CS)	Applicable
GLONASS	L1, L2 CDMA	Secured + FDMA P-code	Prospective

## Data Availability

Dataset available on request from the authors.

## Abbreviations

The following abbreviations are used in this manuscript:

GNSS	Global navigation satellite system
DOA	Direction of arrival
IMU	Inertial measurement unit
PRS	Public regulated service
CASM	Coherent adaptive subcarrier modulation
POCET	Phase-optimized constant-envelope transmission
SCER	Security code estimation and replay
USW	Unpredictable symbol width
TTFAF	Time to first authenticated fix
TBA	Time between authentications

## References

[1] (2001). Vulnerability Assessment of the Transportation Infrastructure Relying on Global Positioning System. Technical Report, John A. Volpe National Transportation Systems Center. https://rosap.ntl.bts.gov/view/dot/8435.

[2] Bhatti J., Humphreys T.E. (2017). Hostile Control of Ships via False GPS Signals: Demonstration and Detection. Navigation.

[3] Mit R., Zangvil Y., Katalan D. Analyzing Tesla’s Level 2 Autonomous Driving System Under Different GNSS Spoofing Scenarios and Implementing Connected Services for Authentication and Reliability of GNSS Data. Proceedings of the 33rd International Technical Meeting of the Satellite Division of The Institute of Navigation (ION GNSS+ 2020).

[4] Kerns A.J., Shepard D.P., Bhatti J.A., Humphreys T.E. (2014). Unmanned Aircraft Capture and Control via GPS Spoofing. J. Field Robot..

[5] Borio D., Gioia C. (2016). A Sum-of-Squares Approach to GNSS Spoofing Detection. IEEE Trans. Aerosp. Electron. Syst..

[6] Neish A., Lo S., Chen Y.H., Enge P. Uncoupled Accelerometer Based GNSS Spoof Detection for Automobiles Using Statistic and Wavelet Based Tests. Proceedings of the 31st International Technical Meeting of The Satellite Division of the Institute of Navigation (ION GNSS+ 2018).

[7] He Y., Zhuang X., Xu B. (2025). Sparse Decomposition-Based Anti-Spoofing Framework for GNSS Receiver: Spoofing Detection, Classification, and Position Recovery. Remote Sens..

[8] Walker P., Rijmen V., Fernández-Hernández I., Bogaardt L., Seco-Granados G., Simón J., Calle D. Galileo Open Service Authentication: A Complete Service Design and Provision Analysis. Proceedings of the 28th International Technical Meeting of the Satellite Division of the Institute of Navigation (ION GNSS+ 2015).

[9] Anderson J.M., Carroll K.L., DeVilbiss N.P., Gillis J.T., Hinks J.C., OHanlon B.W., Rushanan J.J., Scott L., Yazdi R.A. Chips-Message Robust Authentication (Chimera) for GPS Civilian Signals. Proceedings of the 30th International Technical Meeting of The Satellite Division of the Institute of Navigation (ION GNSS+ 2017).

[10] Wang S., Liu H., Tang Z., Ye B. (2021). Binary Phase Hopping Based Spreading Code Authentication Technique. Satell. Navig..

[11] Mina T.Y., Bhamidipati S., Gao G.X. Detecting GPS Spoofing via a Multi-Receiver Hybrid Communication Network for Power Grid Timing Verification. Proceedings of the 31st International Technical Meeting of The Satellite Division of the Institute of Navigation (ION GNSS+ 2018).

[12] OHanlon B.W., Psiaki M.L., Bhatti J.A., Shepard D.P., Humphreys T.E. (2013). Real-Time GPS Spoofing Detection via Correlation of Encrypted Signals: Real-Time Codeless GPS Spoofing Detection. Navigation.

[13] Psiaki M.L., O’Hanlon B.W., Bhatti J.A., Shepard D.P., Humphreys T.E. (2013). GPS Spoofing Detection via Dual-Receiver Correlation of Military Signals. IEEE Trans. Aerosp. Electron. Syst..

[14] Turner M., Chambers A., Mak E., Aguado L.E., Wales B., Dumville M. PROSPA: Open Service Authentication. Proceedings of the 26th International Technical Meeting of the Satellite Division of the Institute of Navigation (ION GNSS+ 2013).

[15] Turner M., Richardson A., Haddon J., Batiste M., Aguado E., Wales B., Dumville M., Bowden R., Togneri P. Galileo Public Regulated Services (PRS) with Limited Key Distribution. Proceedings of the 28th International Technical Meeting of the Satellite Division of the Institute of Navigation (ION GNSS+ 2015).

[16] Tang X., Wang S., Liu J., Wang F. (2023). A NavCom Signal Authentication Scheme Based on Twice Two-Way Satellite Time Transfer. Remote Sens..

[17] Anderson J. (2025). Authentication Security of PRF GNSS Ranging. arXiv.

[18] Space Systems Command (2022). NAVSTAR GPS Space Segment/Navigation User Segment Interfaces.

[19] Partridge M.D., Dafesh P.A. Code Power Measurement Methodology for GPS Block IIR-M and IIF On-orbit Test Procedures. Proceedings of the 14th International Technical Meeting of the Satellite Division of the Institute of Navigation (ION GPS 2001).

[20] Spilker J.J., Orr R.S. Code Multiplexing via Majority Logic for GPS Modernization. Proceedings of the 11th International Technical Meeting of the Satellite Division of the Institute of Navigation (ION GPS 1998).

[21] Dafesh P.A., Nguyen T.M., Lazar S. Coherent Adaptive Subcarrier Modulation (CASM) for GPS Modernization. Proceedings of the 1999 National Technical Meeting of The Institute of Navigation.

[22] Dafesh P.A., Cahn C.R. Phase-Optimized Constant-Envelope Transmission (POCET) Modulation Method for GNSS Signals. Proceedings of the 22nd International Technical Meeting of the Satellite Division of The Institute of Navigation (ION GNSS 2009).

[23] López-Alt A., Tromer E., Vaikuntanathan V. (2017). Multikey Fully Homomorphic Encryption and Applications. Siam J. Comput..

[24] Humphreys T.E. (2013). Detection Strategy for Cryptographic GNSS Anti-Spoofing. IEEE Trans. Aerosp. Electron. Syst..

[25] China Satellite Navigation Office Update on BeiDou Navigation Satellite System. Proceedings of the 12th Meeting of the International Committee on Global Navigation Satellite Systems (ICG-12).

[26] Thoelert S., Steigenberger P., Montenbruck O. (2024). GLONASS-K2 Signal Analysis. GPS Solut..

